# A behavioral and brain imaging dataset with focus on emotion regulation of women with fibromyalgia

**DOI:** 10.1038/s41597-022-01677-9

**Published:** 2022-09-22

**Authors:** Thania Balducci, Jalil Rasgado-Toledo, Alely Valencia, Marie-José van Tol, André Aleman, Eduardo A. Garza-Villarreal

**Affiliations:** 1 Postgraduate Studies Division of the School of Medicine, Medical, Dental and Health Sciences Program, National Autonomous University of Mexico, Mexico City, Mexico; 2 University of Groningen, Department of Biomedical Sciences of Cells and Systems, Cognitive Neuroscience Center, University Medical Center Groningen, Groningen, the Netherlands; 3 Instituto de Neurobiología, Universidad Nacional Autónoma de México campus Juriquilla, Querétaro, Querétaro, México; 4 Instituto Nacional de Salud Pública, Cuernavaca, Morelos Mexico; 5 Shenzhen Key Laboratory of Affective and Social Neuroscience, Center for Brain Disorders and Cognitive Sciences, Shenzhen University, Shenzhen, China

**Keywords:** Emotion, Databases

## Abstract

Fibromyalgia is a chronic condition characterized by widespread pain, as well as numerous symptoms related to central sensitization such as: fatigue, cognitive disturbances, constipation/diarrhea and sensory hypersensitivity. Furthermore, depression and anxiety are prevalent comorbidities, accompanied by emotion processing and regulation difficulties. Although fibromyalgia physiopathology is still not fully understood, neuroimaging research methods have shown brain structural and functional alterations as well as neuroinflammation abnormalities. We believe that open access to data may help fibromyalgia research advance more. Here, we present an open dataset of 33 fibromyalgia female patients and 33 paired healthy controls recruited from a Mexican population. Dataset includes demographic, clinical, behavioural and magnetic resonance imaging (MRI) data. The MRI data consists of: structural (T1- and T2- weighted) and functional (task-based and resting state) sequences. The task was an emotion processing and regulation task based on visual stimuli. The MRI data contained in the repository are unprocessed, presented in Brain Imaging Data Structure (BIDS) format and available on the OpenNeuro platform for future analysis.

## Abstract (summary)

**Table:** 

Measurement(s)	Blood Oxygen Level-Dependent Functional MRI • brain • Emotion
Technology Type(s)	functional magnetic resonance imaging • Magnetic Resonance Imaging • Emotion regulation task
Factor Type(s)	valence • emotion regulation condition
Sample Characteristic - Organism	Homo sapiens
Sample Characteristic - Environment	laboratory environment
Sample Characteristic - Location	Mexico

## Background & Summary

Fibromyalgia is a condition characterized by chronic widespread pain accompanied by physical and psychological symptoms, such as fatigue, cognitive disturbances, depression and anxiety^1^. Fibromyalgia prevalence is estimated to be around 2% in the general population, being more frequent in women than men (3.98 vs 0.01%, respectively)^2^. Despite its relatively low prevalence, the impact on quality of life^3–5^ and the economic burden^6–8^ is high as a consequence of the chronic and disabling symptoms along with the difficulties in diagnosis and treatment^9^.

The difficulties to establish diagnosis and successful treatments reflect in part the poor understanding of the etiology and physiopathology of fibromyalgia. One of the most accepted hypothesis proposes that fibromyalgia symptoms are due to central sensitization which is a hypersensitivity to noxious and non-noxious stimuli mediated by the central nervous system^10^. Indeed, brain structural^11–13^, functional^14–16^ and neuroinflammatory alterations^17,18^ have been found in fibromyalgia.

Structural and functional magnetic resonance imaging (MRI) have shown changes in processing and regulation of pain, which may amplify the pain or reduce the effectiveness of inhibitory mechanisms. Some of these changes include: increased activation of somatosensory cortex, insula, cingulate cortex, and thalamus in response to painful stimuli^19,20^; increased activation of prefrontal cortex, supplementary motor area, insula and anterior cingulate cortex during warm nonpainful stimuli^21^; reduced volume of amygdala, hippocampus, prefrontal cortex, and anterior cingulate cortex^22,23^; reduced activation or altered functional connectivity of areas related to pain inhibition^24,25^; altered neural responses to pain anticipation^26,27^.

Despite the contributions of MRI to the current knowledge on fibromyalgia, many areas of research need further exploration. For instance, the reproducibility of MRI research results has been largely discussed^28–30^, with sample sizes being frequently mentioned as one of the targets to improve power and reproducibility. This applies well for fibromyalgia research, where the sample sizes from fMRI studies are typically between 12 and 40 participants per group. By contributing open datasets such as this one, researchers may be able to overcome the statistical power problem by accumulating datasets with small sample sizes, normalizing variables with methods such as ComBaT^31^, and comparing them to large-scale datasets (i.e. UK BioBank) where normative (healthy) data can be extracted^32^. Another area of opportunity relates to underlying mechanisms and potential clinical applications such as biomarkers. The search for biomarkers benefits from approaches such as meta-analysis and machine learning which also require big sample sizes.

We hereby wish to open our dataset from a cross-sectional case-control study of females with fibromyalgia, created with the aim of studying functional disturbances and their relation with clinical and cognitive processes. Since fibromyalgia is frequently accompanied by psychological symptoms^33^, including emotion processing and regulation disturbances^34–36^, the clinical and cognitive data in our dataset are focused on those domains. The dataset comprises: structural and functional MRI sequences, behavioral data from an emotion processing and regulation task, clinical and psychological measures. We hope this dataset contributes to the study of fibromyalgia by using it for novel analyses or as part of a larger pooled sample.

## Methods

### Participants

We scanned a total of 67 female participants from November 2018 to August 2019. From this total, one participant was eliminated because of an MRI finding suggestive of cysticercosis. Thus, the final sample of the dataset consists of 66 participants: 33 fibromyalgia participants and 33 healthy controls. Demographic characteristics are summarized in Table 1. All participants were right-handed and the two groups were matched with respect to age and years of education. Participants were eligible if they were between 18 and 50 years old, and if they had completed at least elementary school. Participants with fibromyalgia had previously received the diagnosis by a rheumatologist or an internal medicine specialist. To confirm (or exclude controls) the diagnosis of fibromyalgia in the sample, we used the American College of Rheumatology 1990^37^ and 2016^38^ criteria.

**Table 1:** Demographic characteristics of participants on each group.

	FM (n = 33)	HC (n = 33)
Age, years (SD)	41.7 (6.1)	41.5 (6.0)
Education, n (%)		
Elementary	2 (6.1)	1 (3.0)
High school or technical	11 (33.3)	9 (27.3)
Bachelor	13 (39.4)	14 (42.4)
Postgraduate	7 (21.2)	9 (27.3)
Years of study, years (SD)	15.5 (4.0)	16.5 (3.9)
Marital status, n (%)		
Single	9 (27.3)	7 (21.2)
Married/cohabitating	17 (51.5)	21 (63.6)
Divorced/separated	5 (15.1)	4 (12.1)
Widow	2 (6.1)	1 (3.0)
Occupation, n (%)		
Employed	18 (54.5)	24 (72.7)
Unemployed/Housewife	12 (36.4)	8 (24.2)
Student	3 (9.1)	1 (3.0)
Economic status*, median (range)	C+ (AB - D+)	C+ (AB - D)

FM: Fibromyalgia, HC: healthy controls.

*The instrument used was the AMAI rule 8 × 7 created for Mexican homes, where A/B is the highest economic status category and E is the lowest.

Fibromyalgia participants were excluded in case of presence of a major psychiatric disorder (i.e., psychosis, bipolar disorder, obsessive compulsive disorder), cardiovascular disease, neurological illness, other pain conditions when the pain generated by them was higher than the fibromyalgia pain, and use of opioids. Thus, patients with migraine, tension-type headache, systemic lupus erythematosus, or systemic hypertension were excluded. Some of these conditions are prevalent in fibromyalgia, which could reduce generalizability. However, some other prevalent conditions were allowed such as neuropathic pain, irritable bowel syndrome, depression, and anxiety. Additionally, fibromyalgia participants needed to be able to stop taking analgesic or benzodiazepine (rescue-doses) for at least 24 hours before the MRI-session. Exclusion criteria for healthy controls were: any pain, any psychiatric disorder, any cardiovascular, neurologic, inflammatory, autoimmune or rheumatologic illness. Additional exclusion criteria for all participants were: more than three sessions of psychotherapy in the last 12 months or any other intervention that could interfere with emotional regulation (e.g., mindfulness, yoga, counseling); perimenopause, defined as irregular menstrual cycles when previously regular, or less than a year after last menstrual cycle; and any MRI-contraindications. All participants provided written informed consent. The protocol was approved by the Research Ethics Committee of the National Institute of Psychiatry “Ramón de la Fuente Muñiz” in Mexico City, in accordance with the Declaration of Helsinki.

Evaluations for each participant were conducted over two sessions with no more than two weeks in between. During the first session, clinical interviews and scales were applied (See *Clinical and psychological measures* section). During the second session, participants were trained outside the MRI to perform an emotion processing and regulation task and next, they underwent the MRI session.

### MRI data acquisition

Whole brain functional and anatomical images were acquired using a 3.0 T Philips Ingenia MRI scanner with a 32-channel phased array head coil. All sequences were obtained in a single session. The order of the sequences was: (1) resting state (rs-fMRI), (2) T1-weighted (T1w), (3) T2-weighted (T2w), (4) task (task-fMRI). During the scan session participants were fitted with MRI-compatible headphones and goggles.

The rs-fMRI sequences were acquired using a gradient recalled (GE) echo planar imaging (EPI) sequence with the following parameters: dummies = 5, repetition time (TR)/echo time (TE) = 2000/30.001 ms, flip angle (FA) = 75°, matrix = 80 × 80, field of view (FOV) = 240 mm^2^, voxel size = 3 × 3 × 3 mm, slice thickness = 3.0 mm, slice acquisition order = interleaved (ascending), number of slices = 36, phase encoding direction = AP, volumes = 300, duration = 10 min. Participants were instructed to keep their eyes open looking at a fixation cross (presented through the goggles) while thinking of nothing in particular. To prevent participants from falling asleep, their gaze was monitored using an eye-tracking device. When the participants closed their eyes longer than 10 sec, a reminder to stay awake with the eyes opened was given. If a second reminder was needed, the sequence was re-started. After the sequence was re-started, participants were fully awake, according to the observations through the eye-tracker. The database file (*Clinical_fm_66_.xlsx*) at Zenodo^39^ specifies the participants that needed a reminder or to re-start the sequence.

The T1w sequences were acquired using a three-dimensional FFE SENSE sequence, TR/TE = 7.0/3.5 ms, FA = 8°, FOV = 240 mm^2^, matrix = 240 × 240 mm, number of slices = 180, gap = 0, plane = sagittal, slice thickness = 1.0 mm, voxel size = 1 × 1 × 1 mm.

The T2w sequences were acquired using a three-dimensional FFE SENSE sequence, TR/TE = 2.5/0.3 ms, FA = 90°, FOV = 240 mm^2^, matrix = 240 × 240 mm, number of slices = 180, gap = 0, plane = sagittal, slice thickness = 1.0 mm, voxel size = 1 × 1 × 1 mm.

Finally, the task-fMRI sequence were acquired using a GE EPI with the following parameters: dummies = 5, repetition time TR/TE = 2000/30.001 ms, FA = 75°, matrix = 80 × 80, FOV = 240 mm^2^, voxel size = 3 × 3 × 3 mm, slice thickness = 3.0 mm, slice acquisition order = interleaved (ascending), number of slices = 36, phase encoding direction = AP, volumes = 834, duration = 27.8 min. The task was executed using an MRI-compatible response pad (LS-PAIR, Lumina by Cedrus Corp.). Functional and anatomical raw sequences are available at OpenNeuro^40^.

### fMRI task

Participants performed an emotion processing and regulation task^41^. Behavioral results are available at Zenodo^39^. The task required the participants to respond to three different instructions: Attend, Reappraise, and Suppress, for three type of stimuli according to the emotional valence elicited by them: positive, negative and neutral. In total, there were seven conditions: Attend neutral, Attend negative, Attend positive, Reappraise negative, Reappraise positive, Suppress negative and Suppress positive (neutral applied only for the attend condition). The stimuli consisted of pictures from the International Affective Picture System (IAPS)^42^. To build the task, pairs of pictures with similar content were formed and matched on valence and arousal, as well as on activity and complexity of the scene presented. Of each pair, one picture was used for the Regulate condition and one was used for the Attend condition. Because Suppression was not included in the original task, twelve additional pairs of pictures were added using the same criteria (valence, arousal and complexity) as the original set of pairs. Most pictures displayed scenes with humans.

On the instructions, during the Attend condition, participants were asked to observe the pictures and allow themselves to experience any emotional response elicited by the stimuli without trying to manipulate their emotional experience. The Reappraise condition had two variants depending on the valence of the stimuli: *increase* for positive pictures and *decrease* for negative ones. In the case of *increase*, participants were asked to boost the positive emotional experience elicited by the stimuli by reinterpreting the presented picture in a more positive manner (e.g. a girl with a cake is celebrating something very special or they could think on their own child celebrating a good time). In the case of *decrease*, participants were asked to reduce the negative emotion elicited by the stimuli by interpreting it as something distant and not dangerous for them (e.g., a snake picture as part of a safe zoo exhibition) or by changing the meaning of the situation to something less negative (e.g., an accident picture interpreted as a movie scene). For the Suppress conditions (positive and negative), participants were asked to avoid any emotional expression elicited by the stimuli, so that someone watching them would not be able to infer their emotional state.

**Fig. 1:**
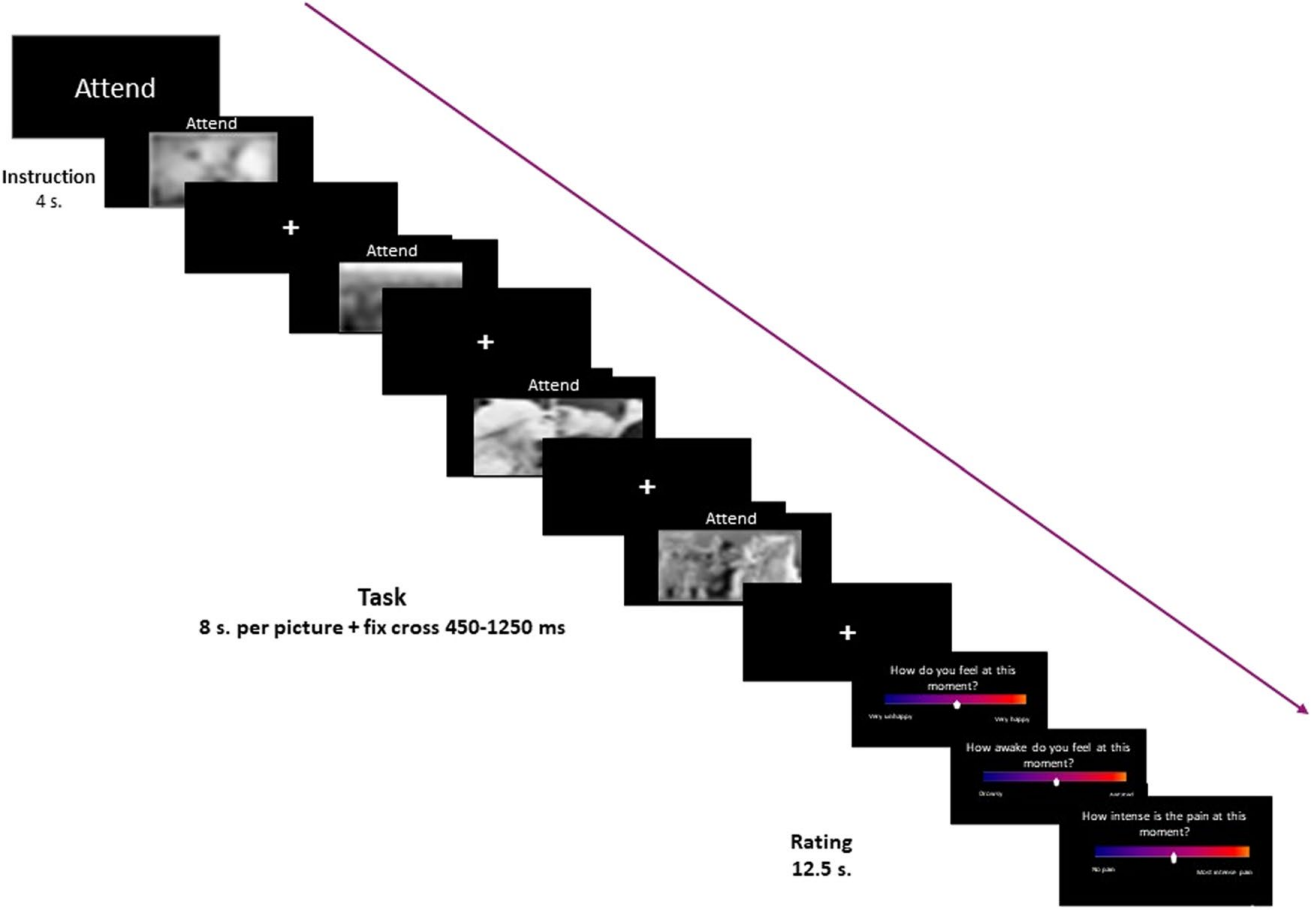
Emotion processing and regulation task. Task design for a single block.

The task was implemented in a block design (Fig. 1). Each condition was repeated three times. Each repetition of a condition was defined as a block. Each block contained the instruction for that block (Attend, Increase, Decrease or Suppress), followed by four pictures, and three evaluation screens with a visual analogue scale on each. Using the visual analogue scales participants rated the intensity and valence of their current affective state (“How do you feel at this moment?”), the arousal (How awake do you feel at this moment?”), and the physical pain at that moment (“How intense is the pain at this moment?”). Scales ranged from 0 to 10. Blocks were presented pseudorandomized (first a neutral block, then six emotional blocks in random order, then seven blocks including both emotional and neutral blocks in random order, then six emotional blocks in random order, and finally a neutral block). Blocks were separated by a fixation cross of varying length (8000 to 14500 ms).

Within each block, the instruction was presented for 4 s, then four pictures during which the same instruction was applied were presented for 8 s per picture. Pictures were separated by a fixation cross, with varying duration (randomized 450 to 1250 ms). After each block of four pictures, the three visual analogue scales were presented consecutively for a maximum of 12500 ms. The task was implemented using E-Prime 3.0.3 software^43^.

Participants were trained outside the MRI to perform the emotional processing and regulation task. First, they were trained to follow the instructions by using a set of pictures taken from the IAPS that were not part of the task. The training was finished when participants were able to give examples of the implementation of the task instructions. Next, using another set of pictures, the participants were trained to perform the task within time constraints and to answer the visual analogue scales using a laptop. Then, participants underwent the MRI session. Before the task-fMRI, participants practiced the use of the response pad. Before and after the scanning, participants rated their fatigue on a visual analogue scale; after the scanning, they rated their performance (ability to execute the instruction) for each task condition on a visual analogue scale. Participants received financial remuneration for their time.

### Preprocessing and first level analysis

#### Task functional data

Preprocessing of the task-fMRI sequences included manual reorientation to the anterior-posterior commissure plane, slice time correction, realignment, co-registration of the functional images to the anatomical images, normalization to Montreal Neurological Institute (MNI) space and smoothing with a full-width at half-maximum Gaussian kernel of 6 mm. These steps were performed using SPM12 (Statistical Parametric Mapping, Wellcome Institute for Cognitive Neurology, London, UK)^44^. Because of a spin-history artifact observed, independent component analysis (ICA) was performed to remove the artifact and other sources of noise. The ICA was executed at the subject-level extracting 100 independent components using the Infomax algorithm, and ICASSO to ensure the stability of components. One researcher (T.B.) selected the components for next analysis manually. The selection of components for 10% of the sample was done by two researchers to test the validity of the selection (T.B., M.J.vT.). The Group ICA of fMRI Toolbox (GIFT v4.0b, MIALab, University of New Mexico, USA) software was used for ICA.

To present the technical validation, data was analyzed in the context of the general linear model, with the time series of each participant convolved with the canonical hemodynamic response function and a 190 s high-pass filter applied. The model included the seven conditions (onsets of each picture and durations) plus the instructions and the visual analogue scales as regressors. Brain activation during fixation was modeled as implicit baseline. Finally, one-sample *t* tests were performed for each instruction (*attend*, *reappraise*, and *suppress*) for each group.

#### Resting state data

Preprocessing was performed using CONN toolbox version 18.b^45^. The pipeline included: realignment, slice timing correction, centering, outlier detection based on Artifact Detection Tools (ART), segmentation, normalization to Montreal Neurological Institute (MNI) space and smoothing (6 mm. full-width at half-maximum Gaussian kernel). Thresholds to tag volumes as outliers were head motion higher than 0.9 mm, and Z score for global signal threshold at 5. Next, denoising was performed. The steps were: linear detrending, outlier censoring, motion regression with 6 subject-specific motion parameters and their first order derivatives, and removal of 10 principal components based on subject-specific white matter and cerebrospinal fluid mask using aCompCor. Finally, a bandpass filter of 0.008 to 0.09 Hz was applied.

## Data Records

Clinical, psychological, behavioral data, and MRI quality assessment data are available at Zenodo^39^ (10.5281/zenodo.6554869). A readme file provides detailed information on the files in the container.

The raw MRI data can be downloaded at OpenNeuro^40^ (https://openneuro.org/datasets/ds004144/versions/1.0.2). We provide scripts and a tutorial to perform the preprocessing of the data (10.5281/zenodo.6554869). Additionally, preprocessed data can be provided upon request.

The MRI data is organized according to the Brain Imaging Data Structure (BIDS, v 1.0.1), and is available on the OpenNeuro Data sharing platform https://openneuro.org/datasets/ds004144/versions/1.0.2. BIDS format facilitates data sharing through the unification of folder structure and file names according to acquisition modality in NIfTI format. BIDS structure includes data descriptions and metadata in JavaScript Object Notation (JSON) files^46^. For our functional files, two labels were used to specify the corresponding task according to BIDS: *rest* for the resting state sequences, and *epr* for the emotion processing and regulation task sequences. Our data were converted from DICOM to NIfTI using dcm2bids through dcm2niix v. 1.0.201^47^. Participants were anonymized by removal of facial features using pydeface v.2.0^48^.

### Clinical and psychological measures

In a session held no more than two weeks prior to the scan-session, participants went through a clinical and psychological evaluation that included: (1) Tender points assessment, (2) Widespread Pain Index and Symptom Severity Scale, (3) Mini International Neuropsychiatric Interview-Plus (MINI-Plus), (Hamilton Depression Rating Scale, (5) Hamilton Anxiety Rating Scale, (5) Emotional Regulation Questionnaire, (6) Toronto Alexithymia Scale, (6) Positive and Negative Affect Schedule, (7) Inventory of Personality Organization, (8) Fibromyalgia Impact Questionnaire, (9) McGill Pain Questionnaire, (10) Fibromyalgia general questionnaire. The assessment was performed by a psychiatrist or a psychologist in a quiet office.

#### Tender points assessment

As a first step to confirm the fibromyalgia diagnosis (and exclude it in controls), we performed a physical exam to evaluate the tender points according to the American College of Rheumatology 1990 diagnosis criteria^37^. The presence of pain on at least 11 of 18 tender points when pressure was applied with a finger over the surface was suggestive of the diagnosis. Painful tender points were registered.

#### Widespread pain index and symptom severity scale

As a second measurement to confirm the fibromyalgia diagnosis (and exclude it in controls), Widespread Pain Index and Symptom Severity Scale were applied. These instruments were introduced in 2010 by the American College of Rheumatology as part of the updated criteria for fibromyalgia diagnosis^49^, and were still valid for the 2016 revision^38^. The Widespread Pain Index is a measure of the number of painful body regions, while the Symptom Severity Scale is a list of 42 symptoms frequently associated with fibromyalgia, with emphasis on tiredness, cognitive symptoms, and unrefreshing sleep.

#### Mini international neuropsychiatric interview-plus

Developed to assess diagnostic criteria for psychiatric disorders according to the Diagnostic and Statistical Manual IV and the International Classification of Diseases 10, the MINI-Plus is a structured interview which was administered by a psychiatrist^50^. We used the official Spanish translation of the version 5.0. The result of this interview is the categorical diagnosis of up to 23 psychiatric disorders.

#### Hamilton depression rating scale

Created by Hamilton in 1960, this scale is still a widely used instrument in clinical and research settings to evaluate the severity of depressive symptoms^51^. The version used was the Spanish translation with 21 items. This is a semi-structured interview and was applied by a psychiatrist or a psychologist. Items are scored in a Likert-type scale from 0 to 4 and the higher the total score the more severe the depression.

#### Hamilton anxiety rating scale

This is an instrument created by Hamilton in 1965 and modified in 1994^52^. Its aim is to evaluate the severity of anxiety symptoms through a semi-structured interview with 14 items, each evaluated in a Likert-type scale from 0 to 4 according to the intensity, frequency and dysfunction caused by the symptoms. The higher the total score the higher the anxiety severity. The Spanish version of the scale was applied by a psychiatrist or a psychologist.

#### Emotional regulation questionnaire

Created by Gross in 2003^53^, this 10-item scale evaluates the use of cognitive reappraisal and expressive suppression, the two most studied regulation strategies and reference for antecedent- and response-focused strategies. The evaluation estimates the efficacy of these strategies to up-regulate positive emotions and down-regulate the negative emotions. In this self-rated instrument, responses are given on a 7-points Likert-type scale.

#### Toronto alexithymia scale

Developed in 1985 and reviewed in 1994, the scale evaluates the personality trait alexithymia, characterized by the difficulty to identify and describe one’s feelings, in addition to an externally oriented thinking style, at the cost of introspection^54^. This is a self-rated scale with 20 items responded in a Likert-type scale. Three factors conform the scale: difficulty to identify emotions, difficulty to express emotions and externally-oriented thought.

#### Positive and negative affect schedule

Created by Watson in 1988, this scale is a self-rated form that evaluates the positive and the negative affective experiences as a trait (usual experience) and as a state (last week). It provides 10 positive and 10 negative adjectives that describe the affect. A rate is assigned to each adjective in a Likert-type scale manner. According to the Mexican validation, the scale presents four factors: positive, shame-guilt, hostility and tension^55^.

#### Inventory of personality organization

Developed by Clarkin in 1995, this self-rating scale intends to measure the level of personality organization according to the model of personality proposed by Kernberg^56^. This model classifies the personality in neurotic, borderline and psychotic depending on the capacity of reality testing, psychological defenses and identity diffusion. The scale has 100 items responded in a Likert-type scale.

#### Fibromyalgia impact questionnaire

Published in 1991, this instrument evaluates the current health status and the severity of symptomatology associated with fibromyalgia. Its 20 items are divided in three sections: everyday activities, capacity to work, and visual analogue scales to evaluate physical and psychological symptoms. The Spanish version has been successfully used for research in the Mexican population previously^57^.

#### McGill pain questionnaire

Developed in 1975, this self-rated scale aims to evaluate the pain from a dimensional perspective which encompass diverse aspects of pain: sensory (temporo-spatial qualities), affective-emotional, evaluative (general description) and miscellaneous. To evaluate these dimensions, a list of 78 adjectives that could describe pain is given, and words are chosen in accordance to the subjective pain experience. The Spanish version of this questionnaire was used in our study^58^.

#### Fibromyalgia general questionnaire

In-house developed set of questions that inquire about the duration and current treatment for fibromyalgia. The questionnaire consisted of three open-ended questions and one close-ended question assessing the duration of fibromyalgia symptoms, the time with the diagnosis, and the current medication received.

## Technical Validation

Quality assessment of the neuroimaging data was performed using MRIQC (v 0.15)^59^, an automated quality control tool that allows to compute and visualize quality metrics such as signal-to-noise ratio, framewise displacement, and other spatial, temporal and artifact parameters.

**Fig. 2:**
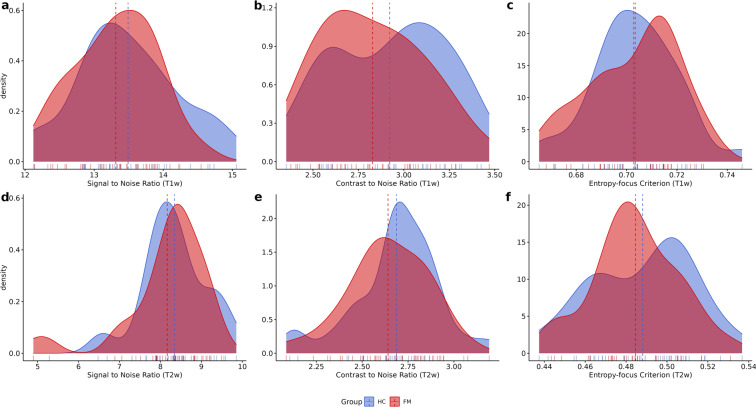
Plots for the quality metrics for structural sequences (Tw1 [upper row] and Tw2 [lower row]). Panel *a* and *d* present the signal-to-noise ratio; panel *b* and *e* display contrast-to-noise ratio; and panel *c* and *f* present the entropy-focus criterion. Dotted lines represent the mean.

**Fig. 3:**
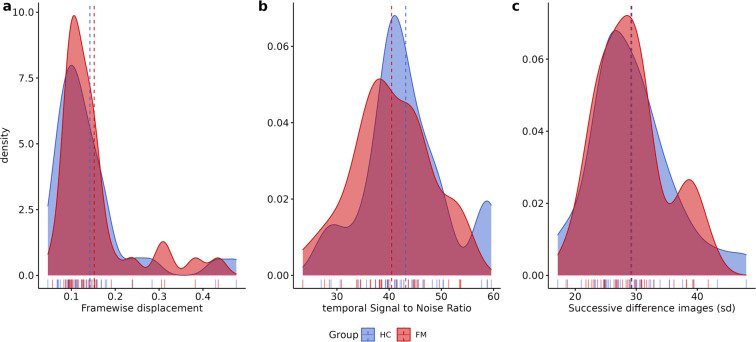
Plots for the quality metrics for resting state functional MRI sequences. Panel (**a**) presents framewise displacement, panel (**b**)shows the temporal signal-to-noise ratio, and panel (**c**) displays the spatial standard deviation of successive difference images. Dotted lines represent the mean.

The quality of MRI sequences was evaluated using the MRIQC v.0.15 assessment. For T1w and T2w here we show: (1) Signal-to-Noise Ratio (SNR) (Fig. 2a,d), (2) contrast-to-noise ratio (Fig. 2b,e) and (3) entropy-focus criterion (Fig. 2c,f). For rs-fMRI images we extracted the following metrics: (1) mean framewise displacement (Fig. 3a), (2) temporal Signal-to-Noise Ratio (Fig. 3b), and (3) spatial standard deviation of successive difference images (DVARS, Fig. 3c).

For the anatomical sequences, SNR is the quotient of the mean signal intensity measured within the tissue mask and the standard deviation of the signal intensity in a region outside the tissue mask^60^. Contrast-to-noise ratio is an extension of SNR for quantitative noise measurement, with the advantage of not being influenced by contrast or brightness changes, where higher values indicate better quality^61^. Entropy-focus criterion is a ghosting and blurring indicator^62^.

For the rs-fMRI, framewise displacement is described as the sum of translational and rotational realignment parameters of instantaneous head motion^63^. Finally, DVARS were calculated to estimate noise variance of functional signals across the brain^64^.

**Fig. 4:**
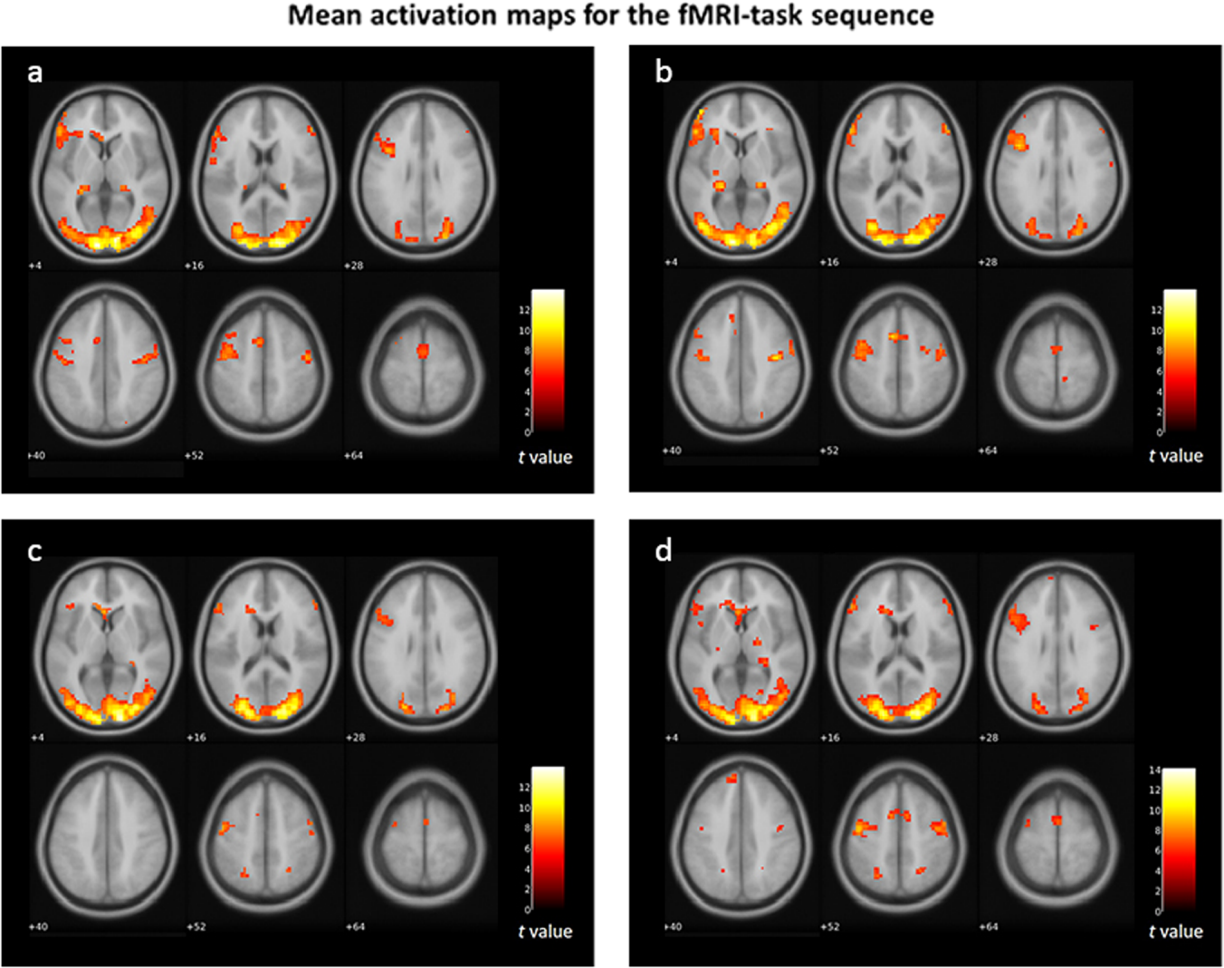
Mean brain activation maps for the task-fMRI sequences. Panel (**a**) mean activation for fibromyalgia participants during *reappraisal*; panel (**b**) mean activation for fibromyalgia participants during *suppression*; panel (**c**) mean activation for healthy control participants during *reappraisal*; panel (**d**) mean activation for healthy control participants during *suppression*.

Besides quality control evaluation through MRIQC, visual assessment of each sequence was performed. An artifact related to spin-history was found in the task-fMRI sequences in some subjects. In this case, quality metrics from MRIQC are not reliable, nevertheless, they are available in Zenodo^39^. Only after removal of the artifact through preprocessing proper quality control can be assessed. Thus, the activation maps that we present for quality control on the task-fMRI sequences represent the data after preprocessing (Fig. 4).

## Usage Notes

The current dataset comprises females with fibromyalgia and healthy controls. The clinical, psychological, behavioral and MRI data can be used for scientific research and academic purposes. Both or one group can be analyzed independently or as part of a larger sample. All data is available in Zenodo and OpenNeuro, accordingly. Due to high motion during scanning, we recommend the use of liberal parameters for framewise displacement and artifacts correction methods; specifically, for the task-fMRI, Independent Component Analysis (ICA) methods are highly recommended to remove the spin-history artifact in the data.

## Data Availability

For the code used for quality control and technical validation, and preprocessing please see the Zenodo repository^39^ (10.5281/zenodo.6554869).
